# RETRACTED ARTICLE: Association of insulin-like growth factor 1 receptor and estrogen receptor with pathological complete response to neoadjuvant chemotherapy in HER2-negative breast cancer

**DOI:** 10.1007/s12282-018-00939-y

**Published:** 2019-01-08

**Authors:** Lei Liu, Yunhui Hu, Sheng Zhang, Xiru Li, Jin Zhang

**Affiliations:** 1 The Third Department of Breast Cancer, China Tianjin Breast Cancer Prevention, Treatment and Research Center, Tianjin Medical University Cancer Institute and Hospital, National Clinical Research Center of Cancer, Huanhu West Road, Tianjin, 300000 China; 2 Department of Surgery, Chinese PLA General Hospital, No.28 Fuxing Road, Beijing, 100071 China

The Editor-in-Chief has retracted this article [1] because, contrary to the statement in the article, the authors have been unable to provide documents confirming that ethics approval was obtained for the study. The ethics committee of Tianjin Medical University Cancer Institute and Hospital has confirmed that it did not approve this study. All authors agree to this retraction.

## Electronic supplementary material

Below is the link to the electronic supplementary material.

Supplementary material 1 (DOC 80 KB)

Supplementary material 2 (PDF 835 KB)
