# Supplementary material for: RETRACTED ARTICLE: Association of insulin-like growth factor 1 receptor and estrogen receptor with pathological complete response to neoadjuvant chemotherapy in HER2-negative breast cancer
Source: Breast Cancer. 2019 Jan 8;26(6):854. doi: 10.1007/s12282-018-00939-y (PMC6821660; doi:10.1007/s12282-018-00939-y)
Supplement: Supplementary file 1 — Supplementary material 1 (DOC 80 KB) [file 12282_2018_939_MOESM1_ESM.doc]

**Supplementary Information**

**Association of insulin-like growth factor 1 receptor and estrogen receptor with pathological complete response to neoadjuvant chemotherapy in HER2-negative breast cancer**

**Running title**: Neoadjuvant chemotherapy in breast cancer

Lei Liu^1^, Yunhui Hu^1^, Sheng Zhang^1^, Xiru Li^2*^, Jin Zhang^1*^

^1^ The Third Department of Breast Cancer, China Tianjin Breast Cancer Prevention, Treatment and Research Center, Tianjin Medical university Cancer Institute and Hospital, National Clinical Research Center of Cancer, Tianjin 300060, China;

^2^ Department of Surgery, Chinese PLA General Hospital, Beijing, 100071, China.

**Corresponding author**: Jin Zhang and Xiru Li

Jin Zhang, Third Department of Breast Cancer, China Tianjin Breast Cancer Prevention, Treatment and Research Center, Tianjin Medical University Cancer Institute and Hospital, National Clinical Research Center of Cancer, Huanhu West Road, Tianjin, 300000, China.

Tel: +8618622221173

Fax: +8618622221173

Email: [zhangjin@tjmuch.com](mailto:zhangjin@tjmuch.com)

Xiru Li, Department of Surgery, Chinese PLA General Hospital, No.28 Fuxing Road, Beijing, 100071, China.

Tel: +8613910594988

Fax: +8613910594988

Email: [2468li@sina.com](mailto:2468li@sina.com)

**Table S1.** Results of **univariate analysis**

| Variable | pCR rate | Chi-square value | *P* value |
| --- | --- | --- | --- |
| Menopausal status at diagnosis |  |  |  |
| Pre | 7.5 | 2.656 | 0.280 |
| Peri | 16.2 |
| Post | 9.8 |
| Tumor size (cm) |  |  |  |
| 2–5 | 10.6 | 0.798 | 0.372 |
| >5 | 7.1 |
| Axillary nodal status |  |  |  |
| Positive | 5.0 | 18.300 |  |
| Negative | 22.2 |  |
| Histological grade |  |  |  |
| Grade 1 | 50.0 | 3.595 | 0.220 |
| Grade 2 | 9.6 |
| Grade 3 | 6.3 |
| TNM stage |  |  |  |
| IIA | 36.7 | 32.785 | <0.001 |
| IIB | 15.2 |
| IIIA | 2.9 |
| IIIB |  |
| IIIC |  |
| Number of chemotherapy cycles |  |  |  |
| 2 | 5.9 | 10.531 | 0.019 |
| 3 | 5.1 |
| 4 | 9.5 |
| 5 | 0 |
| 6 | 50.0 |
| ER |  |  |  |
| Positive | 6.4 | 5.281 | 0.022 |
| negative | 14.9å |
| IGF-1R |  |  |  |
| High expression | 10.4 | 0.191 | 0.662 |
| Low expression | 8.9 |
| HER2 |  |  |  |
| HER2(0) | 0.963 | 0.344-2.692 | 0.943 |
| HER2(1+) | 1.108 | 0.417-2.947 | 0.837 |
| HER2(2+) |  |  | 0.957 |

pCR, pathological complete response; ER, estrogen receptor; IGF-1R, insulin like growth factor 1 receptor; HER2, human epidermal growth factor receptor-2.

**Table S2. The results of logistic regression model**

|  |  |  |  | 95% CI | |
| --- | --- | --- | --- | --- | --- |
| Item | β | Wald Chi-Square | P value | Low | Up |
| Number of chemotherapy cycles |  |  |  |  |  |
| 2 |  | 11.521 | 0.021 |  |  |
| 3 | 0.108 | 0.006 | 0.930 | 0.071 | 17.460 |
| 4 | 1.575 | 1.770 | 0.183 | 0.475 | 49.163 |
| 5 | -17.944 | 0.000 | 0.999 | 0.000 |  |
| 6 | 4.500 | 8.152 | 0.004 | 4.100 | 1976.124 |
| Axillary nodal positive | 0.878 | 1.523 | 0.217 | 0.597 | 9.707 |
| TNM stage |  |  |  |  |  |
| IIA |  | 15.389 | 0.004 |  |  |
| IIB | -1.790 | 6.075 | 0.014 | 0.040 | 0.693 |
| IIIA | -4.275 | 15.230 | 0.000 | 0.002 | 0.119 |
| IIIB | -21.828 | 0.000 | 0.997 | 0.000 |  |
| IIIC | -22.071 | 0.000 | 0.998 | 0.000 |  |
| ER positive | -2.068 | 12.767 | 0.000 | 0.041 | 0.393 |

CI, confidence interval; ER, estrogen receptor.

**Table S3. The relationship between IGF-1R and HER2 expression with IGF1R as a dependent variable**

|  | OR | 95% CI | *P* value |
| --- | --- | --- | --- |
| HER2(0) | 0.745 | 0.409-1.359 | 0.337 |
| HER2(1+) | 0.793 | 0.442-1.423 | 0.437 |
| HER2(2+) |  |  | 0.597 |

HER2, human epidermal growth factor receptor-2; OR, odds ratio; CI, confidence interval.
