# Supplementary material for: RETRACTED ARTICLE: Association of insulin-like growth factor 1 receptor and estrogen receptor with pathological complete response to neoadjuvant chemotherapy in HER2-negative breast cancer
Source: Breast Cancer. 2019 Jan 8;26(6):854. doi: 10.1007/s12282-018-00939-y (PMC6821660; doi:10.1007/s12282-018-00939-y)
Supplement: Supplementary file 2 — Supplementary material 2 (PDF 835 KB) [file 12282_2018_939_MOESM2_ESM.pdf]

Breast Cancer
https://doi.org/10.1007/s12282-018-00939-y

ORIGINAL ARTICLE

Association of insulin-like growth factor 1 receptor and estrogen receptor with pathological complete response to neoadjuvant chemotherapy in HER2-negative breast cancer

Lei Liu[1] · Yunhui Hu[1] · Sheng Zhang[1] · Xiru Li[2] · Jin Zhang[1]

Received: 2 April 2018 / Accepted: 26 November 2018
© The Author(s) 2019

Abstract

Purpose To investigate the association of insulin like growth factor 1 receptor (IGF-1R) and estrogen receptor (ER) with pathological complete response (pCR) secondary to neoadjuvant chemotherapy in human epidermal growth factor receptor-2 (HER2)-negative breast cancer.

Materials and methods The immunohistochemical expressions of IGF-1R and ER were detected in puncture specimens from 273 patients who received neoadjuvant chemotherapy. Association of IGF-1R and ER expression with pCR to neoadjuvant chemotherapy was evaluated.

Results In 273 cases of breast cancer, the high expression rate of IGF-1R was 42.1% (115/273) and the ER-positivity rate was 63.0% (172/273). The positive rate of IGF-1R in ER-positive patients (51.2%) was significantly higher than that in ER-negative patients (26.7%; $p < 0.01$). Multivariate analysis showed that the numbers of chemotherapy circles, TNM stage and ER status were independent factors for pCR rate. The pCR rate in ER-negative patients (14.9%) was significantly higher than that in ER-positive patients (6.4%; $p < 0.05$). The pCR rate in patients with low and high expression of IGF-1R was 8.9% and 10.4%, respectively, which was not statistically significant ($p > 0.05$). Further analysis of the impact of IGF-1R expression status on the pCR rate in ER-positive subgroup did not show any significant associations. Similar results were found in the ER-negative subgroup ($p > 0.05$). In the high-IGF1R subgroup, the pCR rate was higher for ER-negative than that for ER-positive (25.9% vs. 5.7%) patients, which was statistically significant ($p < 0.01$), while in the low-IGF1R subgroup, there was no significant difference between ER-negative and ER-positive (10.8% vs. 7.1%, $p = 0.42$) patients. The pCR rate of ER-negative and high-IGF-1R expression (25.9%) patients was significantly higher than the overall pCR rate ($p < 0.01$).

Conclusion ER-negative patients were more sensitive to neoadjuvant chemotherapy, and the pCR rate was significantly higher in ER-negative than in ER-positive patients in the high-IGF1R subgroup.

Keywords Insulin-like growth factor type 1 receptor · Estrogen receptor · Breast cancer · Neoadjuvant chemotherapy

Electronic supplementary material The online version of this article (https://doi.org/10.1007/s12282-018-00939-y) contains supplementary material, which is available to authorized users.

✉ Xiru Li
240[illegible]i@sina.com

✉ [illegible] Zhang
zh[illegible]jin@tjmuch.com

1 The Third Department of Breast Cancer, China Tianjin Breast Cancer Prevention, Treatment and Research Center, Tianjin Medical University Cancer Institute and Hospital, National Clinical Research Center of Cancer, Huanhu West Road, Tianjin 300000, China

2 Department of Surgery, Chinese PLA General Hospital, No.28 Fuxing Road, Beijing 100071, China

Introduction

Neoadjuvant chemotherapy is the standard treatment for locally advanced breast cancer [1]. It is increasingly used for patients with operable breast cancer who are desirous of breast-conserving surgery [2]. A combination of docetaxel with epirubicin (TE) has been shown to be most effective in reducing the risk of postoperative tumor recurrence and metastasis, and in improving remission rates and prolonging the survival of patients with recurrent metastatic breast cancer [3]. Breast cancer is a highly heterogeneous malignancy at the molecular level, and different subtypes exhibit variable sensitivity to a particular regimen [4]. Identification of subtypes that are highly sensitive to a particular regimen facilitates

the implementation of targeted regimens and individualized treatment.

Pathological complete response (pCR) to neoadjuvant chemotherapy was shown to be an independent predictor of prognosis and to significantly prolong the disease-free survival and overall survival of patients [5]. Response to chemotherapy is influenced by several factors including those related to the breast tumor itself, type of chemotherapy used and the number of treatment cycles [6, 7]. No doubt, the tumor characteristics including tumor size, histological grade, number of involved lymph nodes, and receptor status are equally important. The expression status of several receptors, such as hormone receptors and type 1 insulin-like growth factor receptor (IGF-1R), has been shown to correlate with chemotherapeutic response [8, 9].

IGF-1R is a tyrosine kinase protein that was shown to play an important role in the proliferation, invasion, and metastatic behavior of various cancers including breast cancer [10–13]. IGF-1R expression was shown to be strongly associated with higher mitotic scores and shorter disease-free survival in patients with triple-negative breast cancer [14]. IGF-1R is activated by type 1 insulin-like growth factor (IGF-1) and is related to resistance to chemotherapy and hormonal therapy [15, 16]. Estrogen receptor (ER) is a classical hormone receptor which is a crucial mediator of endocrine therapy for breast cancer. The interaction between IGF-1R and ER has increasingly evoked the interest of researchers. Studies have shown that positive expression of IGF-1R is a favorable prognostic factor in patients with ER-positive tumors; however, this effect is lost in the case of ER-negative invasive ductal breast carcinoma [17]. Zhang et al. [18] demonstrated that IGF1/IGF-1R signaling axis may play a causal role in the resistance of breast cancer cells to anti-estrogen therapy. Indeed, IGF1 is considered a new target for treatment of ER-positive breast cancer [19]. In vitro and in vivo studies have shown that IGF1 signaling pathway induces transcriptional activation of ER target genes and plays an important role in inducing resistance of ER-positive breast cancer to tamoxifen. Moreover, ER can also be activated by the downstream gene of IGF-1R [20, 21].

Therefore, a comprehensive exploration of the correlation of IGF-1R and ER expression status with pCR to neoadjuvant chemotherapy may help in early prediction of the response and to direct the next therapy. However, few studies have investigated this correlation. In this study, we examined the expression levels of IGF-1R and ER proteins in patients with breast cancer to investigate their association with response to TE regimen therapy.

Materials and methods

Patients

This study was approved by the ethical committee of Tianjin Medical University Cancer Institute and Hospital. A retrospective analysis of 273 female patients (age range, 22–69 years) with breast cancer in our hospital from January 2008 to January 2016 was performed. The inclusion criteria were: (1) patients diagnosed with invasive breast cancer by invasive needle biopsy. HER2 evaluated according to immunohistochemical (IHC) examination results [22] with 0–1(+) identified as negative expression; if the level of HER2 was 2 ([illegible]) on [illegible] patient would have to carry on fluorescence in situ hybridization (FISH) detection with HER2 expression identified negative when no amplification was detected; (2) patients with clinical stage (TNM stage) [illegible] III, IIB and IIIA (T3N1M0), who desired breast [illegible] locally advanced disease, including IIIA (T2N2M[illegible] T1N2M0, T2N2M0, T3N2M0), IIIB (T4N[illegible]M0, T4N[illegible]M0, T4N2M0), and IIIC (TN3M0); (3) no history of radiotherapy, chemotherapy or biotherapy; (4) presence of measurable lesions based on the Response Evaluation Criteria In Solid Tumors (RECIST); (5) availability of well-preserved paraffin-embedded specimens for immunohistochemical examination; (6) availability of clinical and imaging data for each cycle of neoadjuvant chemotherapy; (7) availability of pathological data of puncture and postoperative specimens. The clinical and pathological data of 273 breast cancer patients are shown in Table 1.

Treatment

All patients received TE neoadjuvant chemotherapy with docetaxel 75 mg/m^2 plus epirubicin 90 mg/m^2. The responses were evaluated after 2 cycles of chemotherapy to determine the follow-up treatment options. Seventeen patients received 2 cycles, 39 cases received 3 cycles, 199 cases received 4 cycles, 10 cases received 5 cycles, and 8 cases received 6 cycles of chemotherapy. Patients underwent surgery 10–14 days after the end of chemotherapy (6 patients underwent breast-conserving surgery and 267 patients underwent modified radical mastectomy).

Response evaluation

Clinical response to neoadjuvant chemotherapy was evaluated according to RECIST solid tumor assessment criteria (version 1.1) [23]. pCR was defined as absence of invasive

RETRACTED ARTICLE

RETRACTED ARTICLE

Table 1 Patient and tumor characteristics

	N = 473	Percentage (%)
Age at diagnosis (years)		
Mean, range	45 (22–69)	
Menopausal status at diagnosis		
Pre	134	49.1
Peri	37	13.6
Post	102	37.3
Tumor size (cm)		
2–5	189	69.2
>5	84	30.8
Axillary nodal status		
Positive	201	73.6
Negative	72	26.4
Histological grade		
Grade 1	2	0.7
Grade 2	239	85.9
Grade 3	32	11.8
TNM stage		
IIA	30	11.1
IIB	79	28.9
IIIA	103	37.7
IIIB	43	15.8
IIIC	18	6.5

carcinoma in the post-therapy resection specimen and in the regional lymph nodes.

Immunohistochemical examination for IGF-1R and ER expressions

IGF-1R expression is mainly located in the cell membrane or cytoplasm. The following methodology was used for assessment: (1) scores were awarded according to the percentage of positive cells (0 = no positive cells; 1 = 10–25%; 2 = 26–50%; 3 = more than 50% cells); (2) the cell staining intensity score was awarded as follows: 0 = no-coloration; 1 = yellow; 2 = brown; 3 = deep brown. The sum of the above two scores was used to determine the staining intensity level [0–1 = negative (−), 2–3 = weakly positive (+), 4–5 = moderately positive (++), 6 = strong positive (+++)]. The sum of positive cell percentage score and cell staining intensity score more than 4 was classified as high IGF-1R expression and the score less than 4 was classified as low IGF-1R expression. ER-positive standard was at least 10% positive tumor nuclei in the breast samples according to Dutch guidelines (http://www.oncoline.nl).

Statistical analysis

Statistical analysis was performed with SPSS software 22.0 (SPSS, Chicago, IL, USA). pCR was compared among different IGF-1R and ER expression classes using Chi-squared test. Multivariate analysis was done using logistic regression. A p value < 0.05 was considered statistically significant.

Results

ER expression in breast cancer cells is localized in the nucleus. As shown in Fig. 1, nuclear staining present in more than 10% of the cells was considered ER positive. Among 273 breast cancer patients, the positivity rate of ER was 63.0% (Table 2). IGF-1R expression in breast cancer was predominantly seen on the membrane and minimal expression was observed in the cytoplasm (Fig. 2). The high expression rate of IGF-1R was 42.1%. The high expression rate of IGF-1R in ER-positive and ER-negative patients was

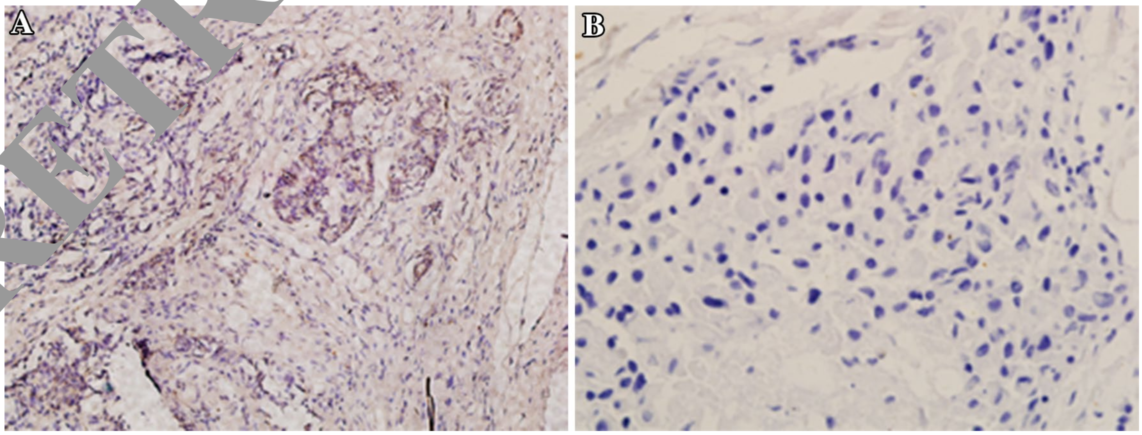

Fig. 1 Representative images of immunohistochemical staining showing **a** ER-positive and **b** ER-negative breast cancer (magnification × 20). *ER* estrogen receptor

RETRACTED ARTICLE

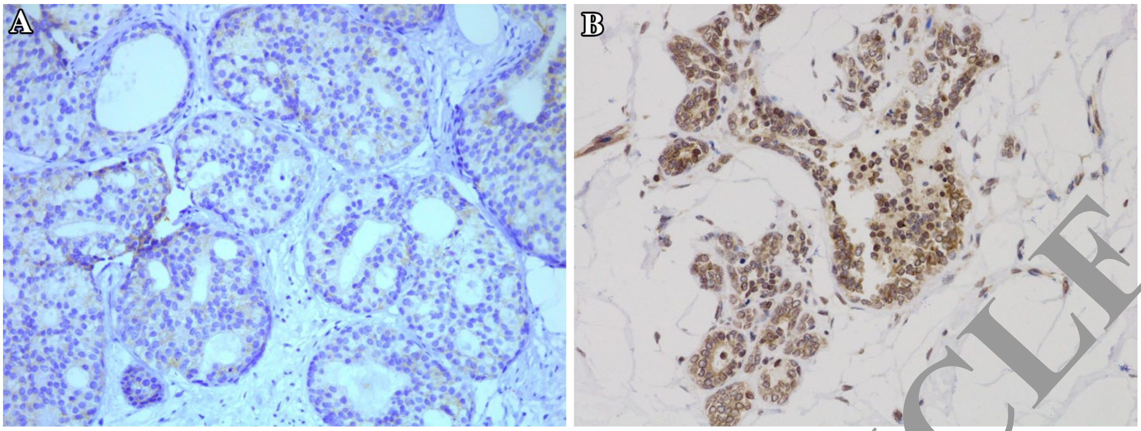

Fig. 2 Representative images of immunohistochemical staining showing a IGF-1R low [illegible] [illegible] ×20) and b IGF-1R high expression (magnification ×40). IGF-1R are stained deep brown. IGF-1R, type 1 insulin-like [illegible] factor [illegible]

Table 2 ER and IGF-1R expression in 273 breast cancer lesions

ER	IGF-1R (%)		[illegible]	Statistical significance (*P*)
	High expression	Low [illegible]		
Positive	88 (51.2)	84 [illegible]	172 (63.0)	< 0.001
Negative	27 (26.7)	74 [illegible]	101 (37.0)	
Total	115 (42.1)	158 [illegible]	273	

ER estrogen receptor, *IGF-1R* [illegible] growth factor receptor 1

51.2% and 26.7%, respectively; the between-[illegible] difference was statistically significant ($p < 0.01$, Table [illegible]

After receiving TE regimen neoadjuvant [illegible], 26 out of the 273 (9.5%) patients showed [illegible] description of different factors is shown in Table S1 and [illegible] analysis results showed that the [illegible] of chemotherapy circles, axillary nodal status, TNM [illegible] and ER status were significant factors for pCR rate. [illegible] analysis showed that the numbers of [illegible] circles, TNM stage and ER status were [illegible] factors for pCR rate (Table S2).

Considering ER [illegible] pCR rate was 14.9% and 6.4% in ER-[illegible] and [illegible] positive patients, respectively; the between-[illegible] difference was statistically significant ($p < 0.05$). [illegible] IGF-1R alone, the pCR rate in patients [illegible] high and low expression levels of IGF-1R was 10.4% [illegible], [illegible]; the between-group difference [illegible] significant ($p > 0.05$).

[illegible] negative subgroup, the pCR rate was numerically [illegible] in high-IGF1R patients than that in low-IGF1R (25.9% vs. 10.8%) patients. However, there was no statistical significance ($p = 0.11$). In the ER-positive subgroup, the PCR rate of high-IGF1R and low-IGF1R patients was 5.7% and 7.1%, respectively. There was no significant difference ($p > 0.05$). In the high-IGF1R subgroup, the pCR rate was higher in ER-negative than in ER-positive (25.9% vs. 5.7%) patients, which was statistically significant ($p < 0.01$). While [illegible] the low-IGF1R subgroup, there was no significant difference between ER-negative and ER-positive (10.8% vs. 7.1%, $p = 0.42$) patients. The PCR rate of ER-negative/high-IGF1R was significantly higher than that of the overall PCR rate ($p < 0.05$) (Table 3).

Discussion

In this study, we analyzed the expressions of ER and IGF-1R in 273 breast cancer patients and found that high expression of IGF-1R was commonly observed in ER-positive cancers and low expression was observed in ER-negative cancers. Our findings are consistent with the results reported by Rohit Bhargava and colleagues [24], and we believe that the expression patterns observed in the present study support the close link between the expressions of IGF-1R and ER. It is evident that IGFs pathways can activate ER, and the activated ER complexes bind to the target genes including IGF-1R and regulate their transcription. An increasing body of evidence from preclinical studies shows that the effects of ER activation may be amplified by IGF-1R with or without estradiol. Therefore, co-targeting ER and IGF-1R may offer benefit to breast cancer patients [25].

Subsequently, we analyzed the association of IGF-1R and ER expression status with pCR to TE regimen neoadjuvant chemotherapy. We found that regardless of IGF-1R

Table 3 Association of ER and IGF-1R with pCR to neoadjuvant chemotherapy in 273 breast cancer patients

	pCR (%)	Not pCR (%)	Statistical significance (*P*)
ER^{+}	11 (6.4)	161 (93.6)	0.02
ER^{-}	15 (14.9)	86 (85.1)	
$IGF\text{-}1R^{+}$	12 (10.4)	103 (89.6)	0.66
$IGF\text{-}1R^{-}$	14 (8.9)	144(91.1)	
ER^{+}, $IGF\text{-}1R^{+}$	5 (5.7)	83 (94.3)	0.94
ER^{+}, $IGF\text{-}1R^{-}$	6 (7.1)	78 (92.9)	
ER^{-}, $IGF\text{-}1R^{+}$	7 (25.9)	20 (74.1)	0.11
ER^{-}, $IGF\text{-}1R^{-}$	8 (10.8)	66 (89.2)	
$IGF\text{-}1R^{+}$, ER^{+}	5 (5.7)	83 (94.3)	0.007
$IGF\text{-}1R^{+}$, ER^{-}	7 (25.9)	20 (74.1)	
$IGF\text{-}1R^{-}$, ER^{+}	6 (7.1)	78 (92.9)	0.42
$IGF\text{-}1R^{-}$, ER^{-}	8 (10.8)	66 (89.2)	
ER^{-}, $IGF\text{-}1R^{+}$	7 (25.9)	20 (74.1)	0.02
All patients	26 (9.5)	247 (90.5)	

ER+ estrogen receptor positive, *ER−* estrogen receptor negative, *pCR* pathological complete response, *IGF-1R*$^{+}$ IGF-1R high expression, *IGF-1R*$^{-}$ IGF-1R low expression

expression, the pCR rate in ER-negative patients was significantly higher than that in ER-positive patients. This result is similar to other published data [26]. Considering IGF-1R expression alone, no significant differences with respect to the pCR rate were observed between patients with low and high expressions of IGF-1R. There is a dearth of studies that have investigated the association between IGF-1R expression alone and the prognosis of breast cancer. Mostly, studies have revealed the association of IGF-1R with shorter disease-free survival in patients with different molecular subtypes of breast cancer, especially in the context of ER-positive and triple-negative breast cancer [15, 17].

We next evaluated the effect of IGF-1R on the pCR of ER-positive and ER-negative patients separately. The results showed that among all the groups, ER-negative and IGF-1R high expression patients exhibited the highest (25.9%) pCR. This result is consistent with the previous literature. Rohit Bhargava et al. [24] analyzed 86 breast cancer patients who received neoadjuvant chemotherapy and found that patients with ER-negative and high IGF-1R expression had the highest sensitivity to the TE regimen (pCR rate: 40%). Further analysis of the impact of IGF-1R expression on the pCR rate in ER-positive and ER-negative subgroups separately showed no significant differences. But Rohit Bhargava et al. [24] found that IGF-1R could be predictive of pCR in the ER-positive subgroup and provided no useful information in the ER-negative subgroup. This difference is likely attributable to differences with respect to the analytic method for immunohistochemistry staining of IGF-1R, sample size and the diversity of the study population between the two studies. In their study, only membranous immunoreactivity was taken into account for scoring of IGF-1R, while in the present study, both membranous and cytoplasmic staining were considered.

There are several limitations of the present study. A previous study found that cytoplasmic IGF-1R was associated with a more favorable prognosis in ER-positive breast cancer; however, this effect was not observed with membranous or overall IGF-1R expression [17, 24]. In the present study, we just analyzed the overall expression including the membranous and cytoplasmic IGF-1R expression; the impact of membranous and cytoplasmic IGF-1R on pCR of breast cancers needs to be separately evaluated in a future study. In addition, the sample size is too small with a lot of deviation and more cases are needed in future work.

In conclusion, patients with ER-negative breast cancer are more sensitive to neoadjuvant chemotherapy with TE regimen, and the pCR rate of ER-negative patients was significantly higher than that of ER-positive patients in the high-IGF1R subgroup. Concomitant assessment of biomarkers of ER and IGF-1R may help predict the efficacy of neoadjuvant chemotherapy and can facilitate selection of personalized chemotherapy regimens for specific populations.

Acknowledgements This work was supported by the Tianjin Natural Sciences Foundation (17JCQNJC09900 to YH) and (15JCYBJC28300 to SZ).

Compliance with ethical standards

Conflict of interest The authors declare that they have no conflict of interest.

OpenAccess This article is distributed under the terms of the Creative Commons Attribution 4.0 International License (http://creativecommons.org/licenses/by/4.0/), which permits unrestricted use, distribution, and reproduction in any medium, provided you give appropriate credit to the original author(s) and the source, provide a link to the Creative Commons license, and indicate if changes were made.

References

1. Rapoport BL, Demetriou GS, Moodley SD, Benn CA. When and how do i use neoadjuvant chemotherapy for breast cancer? Curr Treat Options Oncol. 2014;15:86–98.
2. Untch M, Konecny GE, Paepke S, von Minckwitz G. Current and future role of neoadjuvant therapy for breast cancer. Breast. 2014;23:526–37.
3. Espinosa E, Morales S, Borrega P, Casas A, Madronal C, Machengs I, et al. Docetaxel and high-dose epirubicin as neoadjuvant chemotherapy in locally advanced breast cancer. Cancer Chemother Pharmacol. 2004;54:546–52.
4. Esserman LJ, Berry DA, Cheang MC, Yau C, Perou CM, Carey L, et al. Chemotherapy response and recurrence-free survival in

RETRACTED ARTICLE

neoadjuvant breast cancer depends on biomarker profiles: results from the I-SPY 1 TRIAL (CALGB 150007/150012; ACRIN 6657). Breast Cancer Res Treat. 2012;132:1049–62.
5. Cortazar P, Geyer CE. Pathological complete response in neoadjuvant treatment of breast cancer. Ann Surg Oncol. 2015;22:1441–6.
6. Wei S, Liu L, Zhang J, Bowers J, Gowda GA, Seeger H, et al. Metabolomics approach for predicting response to neoadjuvant chemotherapy for breast cancer. Mol Oncol. 2013;7:297–307.
7. Straver ME, Rutgers EJ, Rodenhuis S, Linn SC, Loo CE, Wesseling J, et al. The relevance of breast cancer subtypes in the outcome of neoadjuvant chemotherapy. Ann Surg Oncol. 2010;17:2411–8.
8. Viale G. Characterization and clinical impact of residual disease after neoadjuvant chemotherapy. Breast. 2013;22(Suppl 2):88–91.
9. Ochnik AM, Baxter RC. Insulin-like growth factor receptor and sphingosine kinase are prognostic and therapeutic targets in breast cancer. BMC Cancer. 2017;17:820.
10. Pollak M. The insulin and insulin-like growth factor receptor family in neoplasia: an update. Nat Rev Cancer. 2012;12:159–69.
11. Singh P, Alex JM, Bast F. Insulin receptor (IR) and insulin-like growth factor receptor 1 (IGF-1R) signaling systems: novel treatment strategies for cancer. Med Oncol. 2014;31:805.
12. Amutha P, Rajkumar T. Role of insulin-like growth factor, insulin-like growth factor receptors, and insulin-like growth factor-binding proteins in ovarian cancer. Indian J Med Paediatr Oncol. 2017;38:198–206.
13. Heidegger I, Massoner P, Sampson N, Klocker H. The insulin-like growth factor (IGF) axis as an anticancer target in prostate cancer. Cancer Lett. 2015;367:113–21.
14. Vilmar A, Santoni-Rugiu E, Cillas JG, Huarriz M, Sorensen JB. Insulin-like growth factor receptor 1 mRNA expression as a prognostic marker in advanced non-small cell lung cancer. Anticancer Res. 2014;34:2991–6.
15. Soljic M, Mrklic I, Tomic S, Omrcen T, Sutalo N, Bevanda M, et al. Prognostic value of vitamin D receptor and insulin-like growth factor receptor 1 expression in triple-negative breast cancer. J Clin Pathol. 2018;71:34–9.
16. Milano A, Dal Lago L, Sotiriou C, Piccart M, Cardoso F. What clinicians need to know about antioestrogen resistance in breast cancer therapy. Eur J Cancer. 2006;42:[illegible]692–705.
17. Hartog H, Horlings HM, van der Vegt [illegible], Kreike B, Ajouaou A, van de Vijver MJ, et al. Divergent effects of insulin-like growth factor-1 receptor expression on prognosis of estrogen receptor positive versus triple negative invasive ductal breast carcinoma. Breast Cancer Res Treat. 2011;129:725–36.
18. Zhang Y, Moerkens M, Ramaiahgari S, de Bont H, Price L, Meerman J, et al. Elevated insulin-like growth factor 1 receptor signaling induces antiestrogen resistance through the MAPK/ERK and PI3K/Akt signaling routes. Breast Cancer Res. 2011;13:R52.
19. Zhang M, Hu Z, Huang J, Shu Y, Dai J, Jin G, et al. A 3′-untranslated region polymorphism in IGF1 predicts survival of non-small cell lung cancer in a Chinese population. Clin Cancer Res. 2010;16:1236–44.
20. Tokunaga E, Kimura Y, Mashino K, Oki E, Kataoka A, Ohno S, et al. Activation of PI3K/Akt signaling and hormone resistance in breast cancer. Breast Cancer. 2006;1[illegible]7–44.
21. Jeng MH, Yue W, Eischeid A, Wang JP, Santen RJ. Role of MAP kinase in the enhanced cell proliferation of long term estrogen deprived human breast cancer cells. Breast Cancer Res Treat. 2000;62:167–75.
22. Wolff AC, Hammond ME, Hicks DG, Dowsett M, McShane LM, Allison KH, et al. Recommendations for human epidermal growth factor receptor 2 testing in breast cancer: American Society of Clinical Oncology/College of American Pathologists clinical practice guideline update. J Clin Oncol. 2013;31:3997–4013.
23. Eisenha[illegible]erasse P, Bogaerts J, Schwartz LH, Sargent D, For[illegible], et al. New response evaluation criteria in solid tumours: revised RECIST guideline (version 1.1). Eur J Cancer. 20[illegible]47.
24. Bhargava R, Beriwal S, McManus K, Dabbs DJ. Insulin-like growth factor receptor-1 (IGF-1R) expression in normal breast, proliferative breast lesions, and breast carcinoma. Appl Immunohistochem Mol Morphol. 2011;19:218–25.
25. Fagan DH, Yee D. Crosstalk between IGF1R and estrogen receptor signaling in breast cancer. J Mammary Gland Biol Neoplasia. 2008;13:423–9.
26. Bhargava R, Beriwal S, Dabbs DJ, Ozbek U, Soran A, Johnson RR, et al. Immunohistochemical surrogate markers of breast cancer molecular classes predicts response to neoadjuvant chemotherapy: a single institutional experience with 359 cases. Cancer. 2010;116:1431–9.

RETRACTED ARTICLE
